# *In Silico* Identification of Potent Pancreatic Triacylglycerol Lipase Inhibitors from Traditional Chinese Medicine

**DOI:** 10.1371/journal.pone.0043932

**Published:** 2012-09-06

**Authors:** Kuan-Yu Chen, Su-Sen Chang, Calvin Yu-Chian Chen

**Affiliations:** 1 Department of Chinese Medicine, China Medical University, Taichung, Taiwan; 2 Laboratory of Computational and Systems Biology, China Medical University, Taichung, Taiwan; 3 Department of Medical Research, China Medical University Hospital, Taichung, Taiwan; 4 Department of Biotechnology, Asia University, Taichung, Taiwan; 5 Department of Biomedical Informatics, Asia University, Taichung, Taiwan; 6 China Medical University Beigang Hospital, Yunlin, Taiwan; Consiglio Nazionale delle Ricerche, Italy

## Abstract

Pancreatic triacylglycerol lipase (PNLIP) are primary lipases that are critical for triacylglyceride digestion in human. Since reduced metabolism of triacylglyceride might be a plausible concept for weight loss, we screened for potential PNLIP inhibitors from traditional Chinese medicine (TCM) with the aim to identify weight loss candidate compounds. TCM candidates Aurantiamide, Cnidiadin, and 2-hexadecenoic acid exhibited higher Dock Scores than the commercial drug Orlistat, and were also predicted to have inhibitory characteristics against PNLIP using constructed MLR (R^2^ = 0.8664) and SVM (R^2^ = 0.9030) models. Molecular dynamics indicated that the TCM-PNLIP complexes formed were stable. We identified that the PNLIP binding site has several residues that can serve as anchors, and a hydrophobic corridor that provides additional stability to the complex. Aurantiamide, Cnidiadin, and 2-hexadecenoic acid all have features that correspond to these binding site features, indicating their potential as candidates for PNLIP inhibitors. The information presented in this study may provide helpful insights to designing novel weight-control drugs.

## Introduction

Obesity is a worldwide health issue of increasing importance and is an important risk factor for many other diseases [1]–[4]. It is projected that by 2015, more than 1.5 billion people will be over-weight, and that at least 2.6 million annual deaths can be attributed to obesity [5]. Obesity is a huge burden on social costs and is linked to many chronic diseases and cancer, Pancreatic triacylglycerol lipase (PNLIP) are the primary lipases secreted by the pancreas, and is responsible for breaking down dietary lipids into unesterified fatty acids (FAs) and monoglycerides (MGs). The unesterified FAs and MGs will combine with bile salt, cholesterol, and lysophosphatidic acid (LPA) to form micelles. Once absorbed by the intestines, it will be re-synthesized to triacylglycerides and stored within the lipid cells as a major source of energy for the human body. Since ingesting too much dietary lipids equals excessive calorie intake, targeted inhibition of PNLIP may reduce caloric intake and have implications in weight control [6]–[8].

Orlistat is a weight-loss drug that reduces lipid adsorption through the inhibition of PNLIP [9], [10]. However, it can only reduce approximately 30% lipid adsorption. Since these lipids are excreted from the body through stool excrements, major side-effects of Orlistat involve gastrointestinal tract issues [11]. Long term use of Orlistat also interferes with the adsorption of lipid-soluble vitamins. This research primarily focuses on identifying inhibitors of PNLIP in hopes of providing better alternatives for obese patients.

Conventional drug design is a labor-intensive, resource-taxing, and time-consuming process with low success rates. To accelerate drug research, reduce research costs and improve success rates, computer-aided drug design (CADD) is currently becoming an important means of designing new drugs [12]. Many studies have reported the potential application of TCM compounds in allergy, cancer, diabetes, influenza, and stroke, etc [13]–[20]. Based on the need for rapid screening and to provide access to the largely untapped resources of traditional Chinese medicine (TCM), the Traditional Chinese medicine Database@ Taiwan (http://tcm.cmu.edu.tw/) [21] and its cloud-computing server iScreen (http://iscreen.cmu.edu.tw/) [22] and iSMART [23] were developed. This research utilizes TCM Database@Taiwan to screen for compounds that demonstrate drug like characteristics against PNLIP to provide inspiration for developing novel PNLIP inhibitors.

## Results and Discussion

### Docking and screening

**Figure 1:**
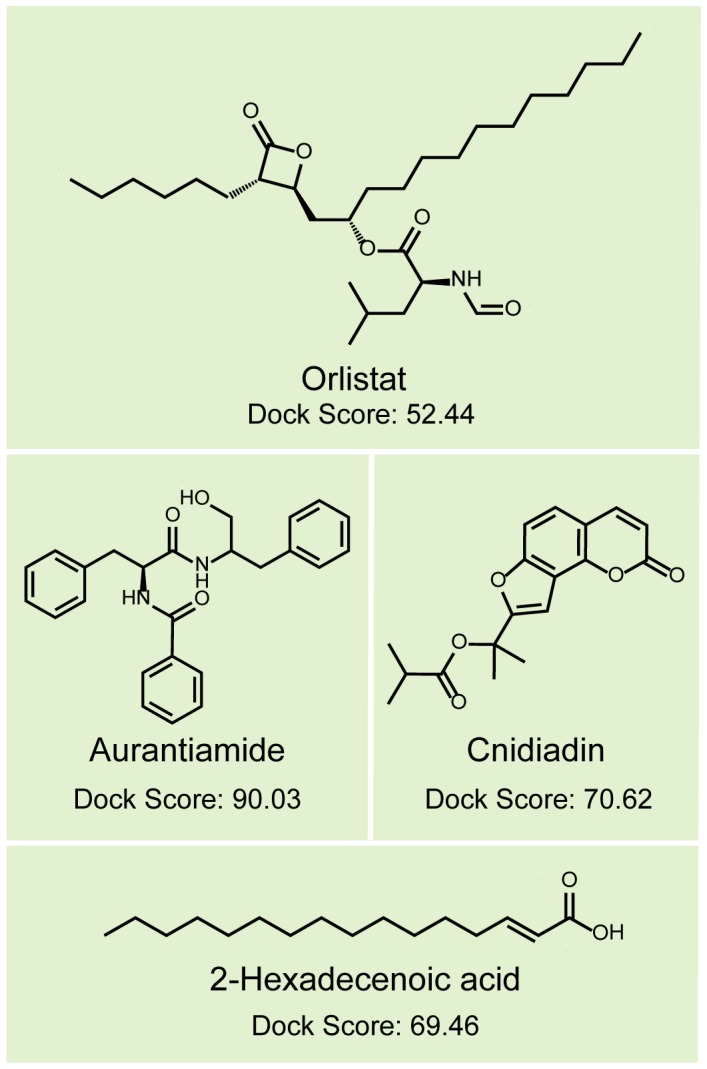
Structural scaffolds and Dock Scores of the top ten TCM compounds from TCM Database@Taiwan. Candidate compounds investigated further in this study are highlighted with the dark green background in addition to the control compound Orlistat.

**Figure 2:**
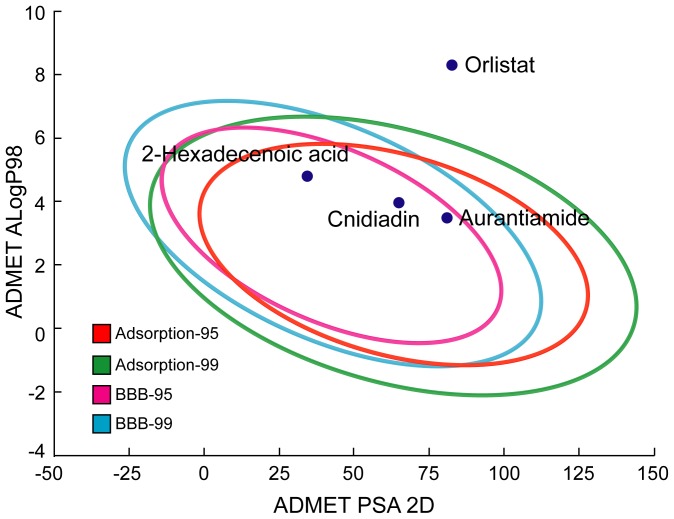
Adsorption model of the candidate compounds.

**Figure 3:**
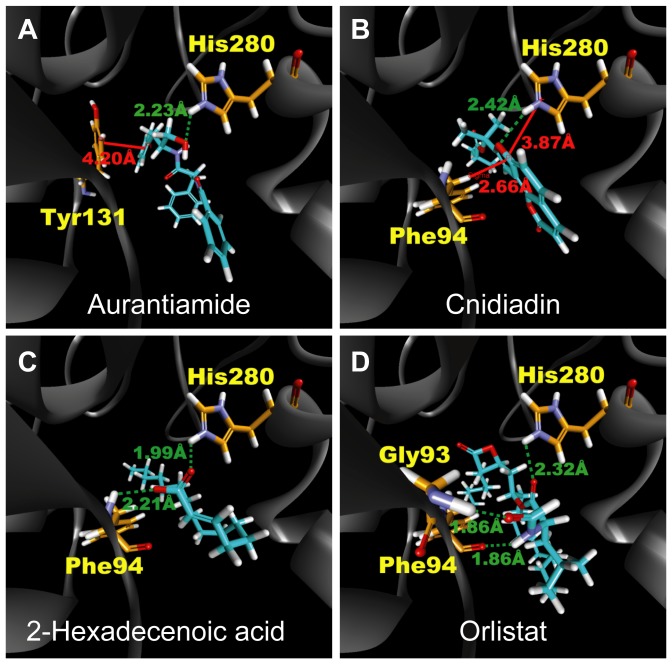
Docking poses of test ligands within PNLIP binding site. (A) Aurantiamide, (B) cnidiadin,(C) 2-hexadecenoic acid, and (D) Orlistat. Residues on which interactions are formed are labeled in yellow. Green dash lines and red solid lines depict H-bonds and pi-interactions, respectively. Corresponding distances of the interactions are also given.

TCM compounds aurantiamide, cnidiadin, and 2-hexadecenoic acid, were selected as candidates based on their high Dock Score compared to Orlistat (Figure 1). These candidates should be more easily adsorbed by the human body than Orlistat as indicated by the adsorption and blood brain barrier properties (Figure 2). Aurantiamide docking within PNLIP binding site was maintained by a pi interactions with Tyr131 and a hydrogen bond (H-bond) with His280 (Figure 3A). Affinity between Cnidiadin and PNLIP can be attributed to the pi interaction with Phe94 and the H-bond and pi interaction with His280 (Figure 3B). Identically, 2-hexadecenoic acid also interacted with Phe94 and His280 through H-bonds (Figure 3C). Orlistat, the control drug, formed H-bonds with Gly93, Phe94, and His280 (Figure 3D). The docking poses of TCM candidates resembled that of Orlistat, each interacting with His280 and either Phe94 or Tyr131. Based on these results, Phe94 and His280 are important for ligand-PNLIP interactions.

### Multiple linear regression (MLR) and support vector machine (SVM) model construction and bioactivity prediction

**Figure 4:**
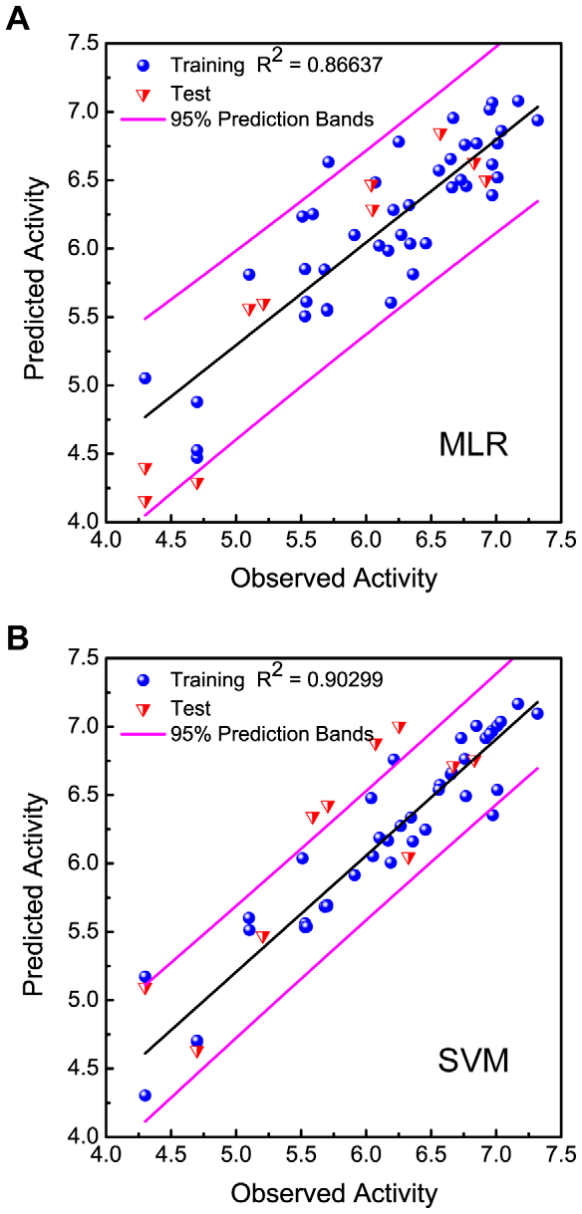
Correlation between predicted and observed bioactivities (pIC_50_) using the (A) MLR and (B) SVM models.

The ten representative genetic descriptors for bioactivity determined by Genetic Function Approximation (GFA) are: *ALogP_MR*, *CIC*, *IC*, *Jurs_FPSA_2*, *Jurs_RNCS*, *Jurs_RPCG*, *Jurs_WPSA_3*, *RadOfGyration*, *Shadow_Yzfrac*, *Shadow_Zlength*. Using these descriptors, the following MLR and SVM prediction models were constructed. The constructed MLR model is:
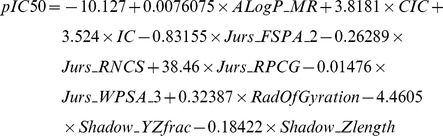



Based on the square correlation coefficients calculated for MLR (R^2^ = 0.8663; Figure 4A) and SVM (R^2^ = 0.9029; Figure 4B), the two models are reliable. Predicted bioactivities of the top ten TCM compounds using the two validated models are listed in Table 1. Results imply that the TCM compounds have high bioactivity against PNLIP.

**Table 1:** Predicted pIC_50_ of top tenTCM ligands against PNLIP by MLR and SVM models.

Name	Predicted pIC_50_	
	MLR	SVM
Aurantiamide	6.910	6.043
Cnidiadin	8.046	5.785
2-Hexadecenoicacid	8.015	5.994
6-Hydroxy-8-shogaol	5.920	6.119
8-Hydroxy-9-E-octadecenoicacid	6.584	6.166
10-Hydroxy-8-E-octadecenoicacid	6.833	6.337
2-Pentadecenoicacid	7.877	5.866
Piperanine	6.748	5.355
9,12-Octadecadienoicacid	7.582	6.292
1,3-Cyclopent-2-enyltridec-4-enoicacid	7.826	6.331
Orlistat *	7.606	7.073

### Molecular dynamics (MD) simulation

**Figure 5:**
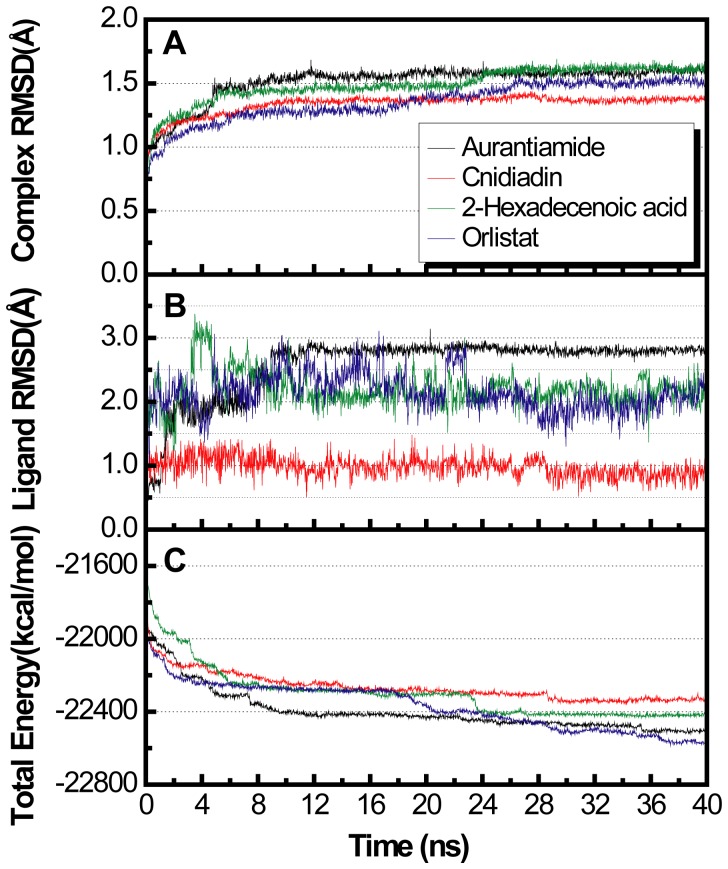
MD trajectories depicting changes during 40 ns simulation. (A) Plot of complex RMSD, (B) plot of ligand RMSD, and (C) plot of complex total energy verses MD simulation time.

RMSD and total energy of the test ligands stabilized with time and achieved equilibrium by the end of MD (Figure 5). Compared to Orlistat, complexes RMSDs of the TCM candidates stabilized faster, a phenomenon likely due to their compact structure (Figure 5A). Stability of individual ligands within the binding site are shown in Figure 5B. Cnidiadin was most stable, maintaining a ligand RMSD of approximately 1 Å. Other compounds exhibited larger RMSD and fluctuations. Total energies of complexes following equilibrium were Cnidiadin>2-hexadenoic acid>Aurantiamide>Orlistat (Figure 5C).

**Figure 6:**
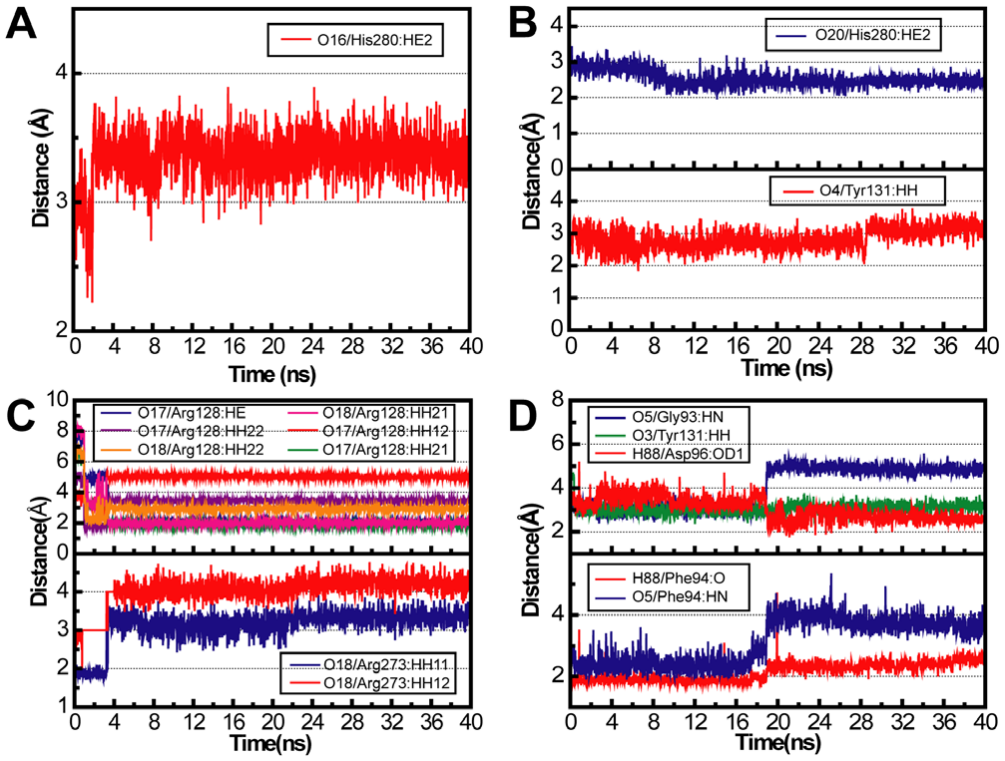
Distance of H-bonds formed between test compounds and PNLIP binding site during MD. (A) Aurantiamide, (B) Cnidiadin, (C) 2-hexadecenoic acid, and, (D) Orlistat.

Weak forces such as H-bonds and hydrophobic interactions play critical roles in the ligand recognition and protein stability, and were analyzed separately. Table 2 summarizes H-bond formation and stability during MD. Aurantiamide formed a single low occupancy H-bond with His280. Cnidiadin formed H-bonds with Tyr131 (17.85%)_and His280 (50.75%). 2-Hexadecenoic acid primarily interacted with Arg128 and Arg273 during MD. Three H-bonds with Arg128 were highly stable with occupancies greater than 91.50%. Orlistat formed stable H-bonds with Gly93, Phe94, Asp96, and Tyr131. Based on the occupancy rate and observation frequency, Phe94 was the key residue for H-bond formation. To account for possible underestimation of H-bond occupancies due to the designated cutoff distance 2.5 Å, H-bond distance trajectories of each individual H-bond were analyzed. Based on the trajectory shown in Figure 6A, the H-bond distance with His280 generally exceeded the typical H-bond distance of 2.2–3.2 Å, indicating that Aurantiamide was probably stabilized within the complex by interactions other than H-bonds. For Cnidiadin, H-bonds at Tyr131 and His280 were within typical H-bond distance ranges (Figure 6B), implying that H-bonds formed at these locations were stable and effectual in maintaining stability during MD. H-bond trajectories for 2-hexadecenoic acid at Arg128 (Figure 6C) show consistent findings to those in Table 2. All six H-bonds detected at Arg128 may contribute to stability albeit some distance being greater than 2.5 Å. The primary H-bonds formed by Orlistat were with Phe94, Asp96, and Tyr131 (Figure 6D). Initially, a weak H-bond was formed with Gly93, but was substituted by that with Asp96 at the end of MD. This substitution could be due to conformational changes that increase the distance from Gly93 and decrease the distance from Asp96. Overall, H-bonds were important for the stability of Cnidiadin, 2-hexadecenoicacid, and Orlistat.

**Table 2:** H-bond interactions between PNLIP and top three candidates and Orlistat.

Ligand	H-bond	Ligand Atom	Amino acid	Distance (Å)			H-bond occupancy
				Max.	Min.	Avg.	
Aurantiamide	1	O16	His280:HE2	3.893	2.221	3.347	0.35%
Cnidiadin	1	O20	His280:HE2	3.447	1.963	2.534	50.75%
	2	O4	Tyr131:HH	3.779	1.831	2.829	17.85%
2-Hexadecenoicacid	1	O17	Arg128:HE	7.949	1.782	2.377	91.50%
	2	O17	Arg128:HH12	5.452	2.150	4.880	0.30%
	3	O17	Arg128:HH21	6.955	1.585	2.056	91.85%
	4	O18	Arg128:HH21	8.409	1.679	2.246	91.80%
	5	O17	Arg128:HH22	5.414	1.606	3.394	5.65%
	6	O18	Arg128:HH22	7.191	1.846	2.992	4.50%
	7	O18	Arg273:HH11	3.910	1.680	3.123	8.50%
	8	O18	Arg273:HH12	4.806	2.460	4.018	0.10%
Orlistat	1	H88	Phe94:O	4.733	1.643	2.141	88.30%
	2	O5	Phe94:HN	5.353	1.850	4.031	31.85%
	3	O5	Gly93:HN	5.576	2.391	3.137	0.25%
	4	O3	Tyr131:HH	4.712	2.366	3.065	0.25%
	5	H88	Asp96:OD1	5.204	1.745	3.051	13.80%

H-bond occupancy cutoff: 2.5 Å.

**Figure 7:**
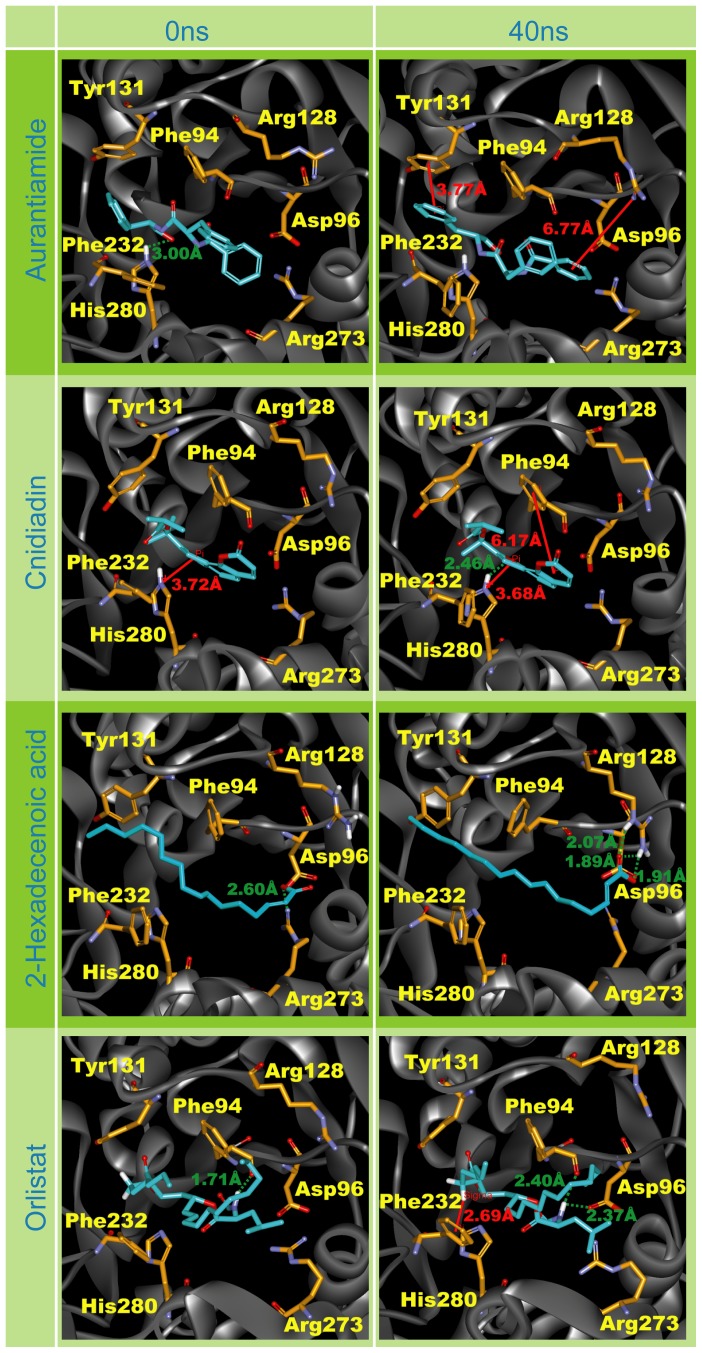
Ligand interaction changes during MD. Snapshots are taken for each test compound at 0 ns and 40 ns.

MD snapshots of the test compounds at 0 ns and 40 ns may help visualize interactions involved in PNLIP-ligand complex stability (Figure 7). As previously mentioned, H-bonds were not a primary stabilizing factor for aurantiamide. The snapshots at 0 ns and 40 ns support this view. At the end of MD, Aurantiamide was anchored within the binding site by pi-interactions with Arg128 and Tyr131 while no H-bonds were observed. Pi-interactions were also involved in stabilizing Cnidiadin during MD. The two pi-interactions on opposing sides of Cnidiadin served as invisible chains to anchor Cnidiadin within the PNLIP binding site. These interactions may greatly inhibit ligand movement and contribute to the stable ligand RMSD in Figure 5B. 2-Hexadecenoic acid did not form pi-interactions, and was stabilized through its hydrophilic head region by multiple H-bonds with Arg128 and Arg273. Initially, Orlistat formed only H-bonds, but upon complex stabilization, an additional pi-interaction with Phe232 was observed.

**Figure 8:**
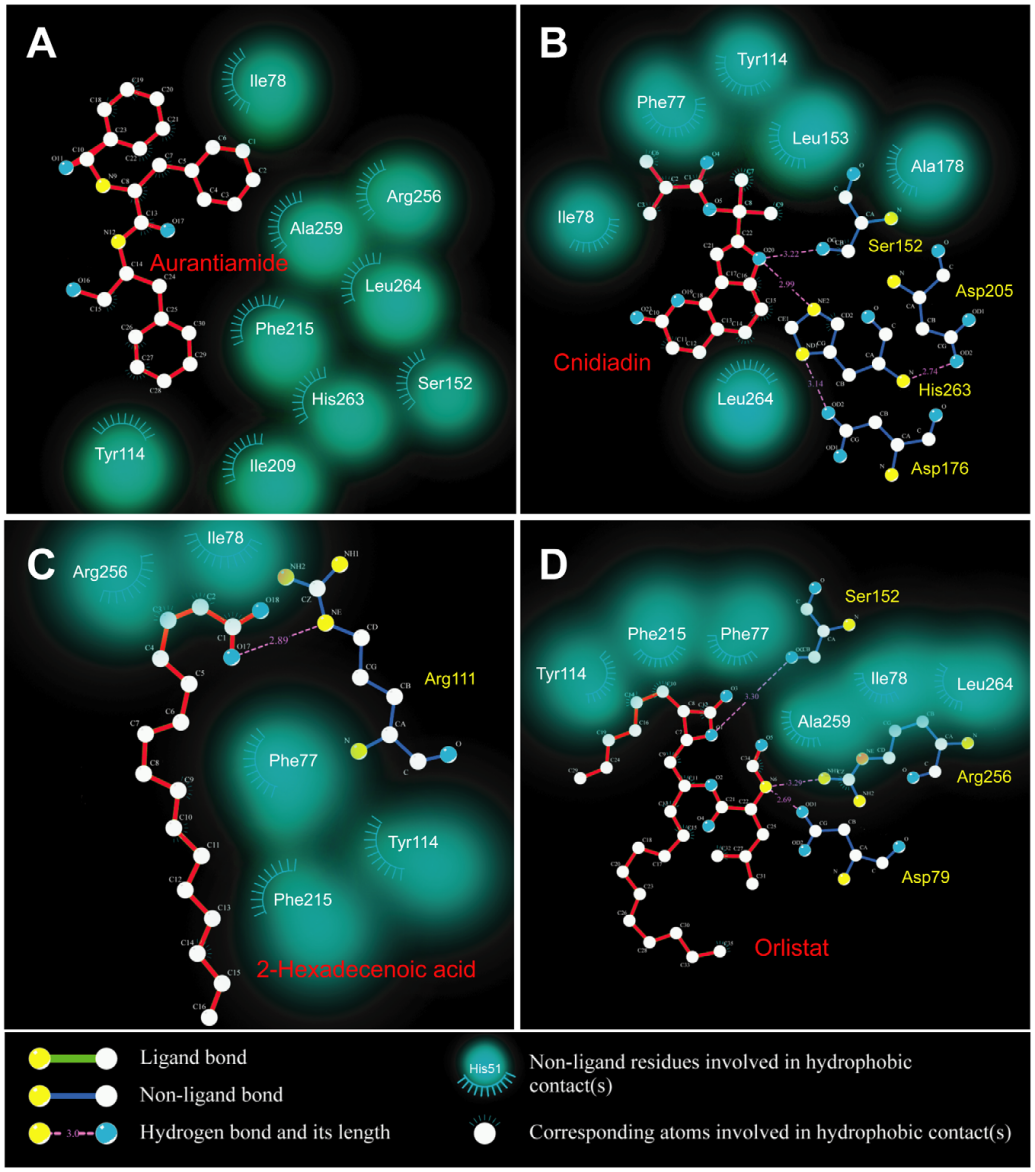
Ligplot diagrams illustrating protein-ligand interactions. (A) Aurantiamide, (B) Cnidiadin, (C) 2-Hexadecenoic acid, (D) Orlistat.

Total hydrophobic interactions are also critical for stabilization and the results of Ligplot analysis are shown in Figure 8. The highest number of hydrophobic interactions was observed in Aurantiamide (Figure 8A). This was expected as Aurantiamide lacked H-bonds compared to the other test compounds. The hydrophobic contacts primarily interacted with the end cyclohexanes and carbon backbone, providing additional support in addition to the pi-interactions with Arg128 and Tyr131. This balance between interaction forces secures Aurantiamide within the binding site and may be the reason for its low total energy (Figure 5C). Six hydrophobic contacts were formed with Cnidiadin and served to stabilize side chains that were not bound by H-bonds and pi-interactions (Figure 8B). Hydrophobic contacts were responsible for stabilizing the aliphatic tail of 2-hexadecenoic acid (Figure 8C). However, not all C-atoms on the tail formed hydrophobic contacts, thus some flexibility of the tail was still retained. The hydrophobic contacts formed with Orlistat primarily interacted with the carbon backbone, providing stabilizing forces on the backbone which was not restricted by pi or H-bonds (Figure 8D). The ability of residues Ile95/Tyr131 to form hydrophobic contacts with all compounds, and the ability of Phe94/Phe226/Arg232 to form hydrophobic contacts with three test compounds imply that they may play major roles in ligand-PNLIP stability. Hydrophobic contacts formed by the TCM candidates were similar to those by Orlistat; Aurantiamide shared all hydrophobic contacts formed by Orlistat except for Phe94, and Cnidiadin and 2-hexadecenoic acid shared four out of the six in Orlistat.

**Figure 9:**
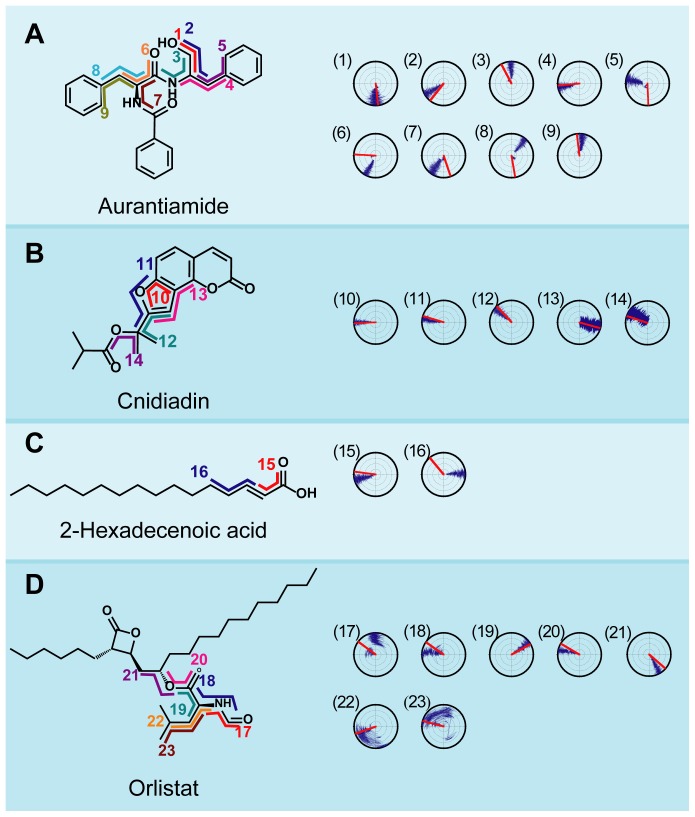
Torsion angle changes during 40 ns MD simulation. Each torsion angle is specified by a numerical and corresponds to the radar chart with the identical number. (A) Aurantiamide, (B) Cnidiadin, (C) 2-Hexadecenoic acid, (D) Orlistat.

Important information regarding molecular stability can also be obtained through torsion angle changes. Positions where H-bond or pi-interactions formed were monitored for torsion changes throughout MD (Figure 9). Torsion changes for aurantiamide were small with the exception of 1 and 5 (Figure 9A). Cnidiadin was stable with small torsion changes due to its relatively rigid structure and stabilizing forces described previously (Figure 9B). Torsion changes observed for the H-bond forming hydrophilic head region were also limited (Figure 9C). The largest amount of torsion angle fluctuation was observed in Orlistat at positions **17**, **22**, and **23** (Figure 9D). From a structural view, **22** and **23** were the end points of a carbon chain, and can more easily have irregular fluctuations leading to large torsions. The fluctuations observed at 17 corresponded to the H-bond stabilization at this location (Figure 6D). The two distinct groups of the torsion angles was the result of H-bond distance fluctuations between 16–20 ns. In general, the H-bonds and pi-interaction formed by the candidates were stable, limiting changes in torsion angles.

**Figure 10:**
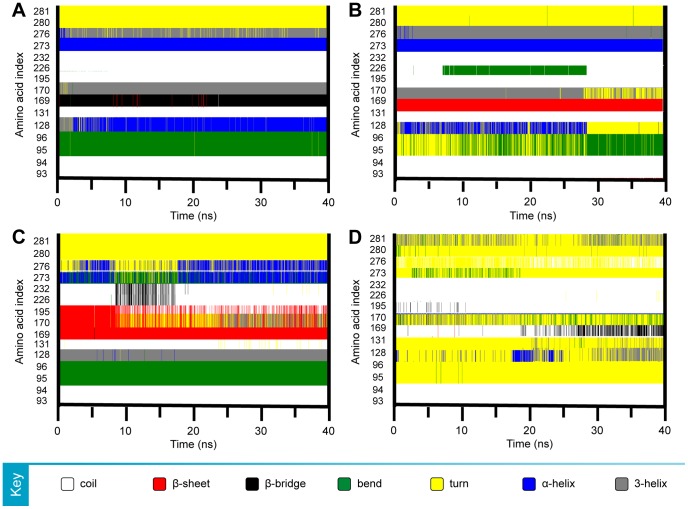
Secondary structure changes observed for binding cleft amino acid residues during the 40 ns MD simulation. (A) Aurantiamide, (B) Cnidiadin, (C) 2-Hexadecenoic acid, (D) Orlistat.

Insights to structural changes that contribute to stability differences were observed by secondary structure changes (Figure 10). Aurantiamide (Figure 10A) and cnidiadin (Figure 10B) exhibited similarly stable secondary structure. Cnidiadin formed a stable β-sheet as opposed to the β-bridge in Aurantiamide. In PNLIP-2-hexadecenoic acid complex, disruption rather than the formation of stable structures was observed. The PNLIP-Orlistat complex consisted of primarily turns or coils, and lacked the presence of α-helixes or β-sheets.

**Figure 11:**
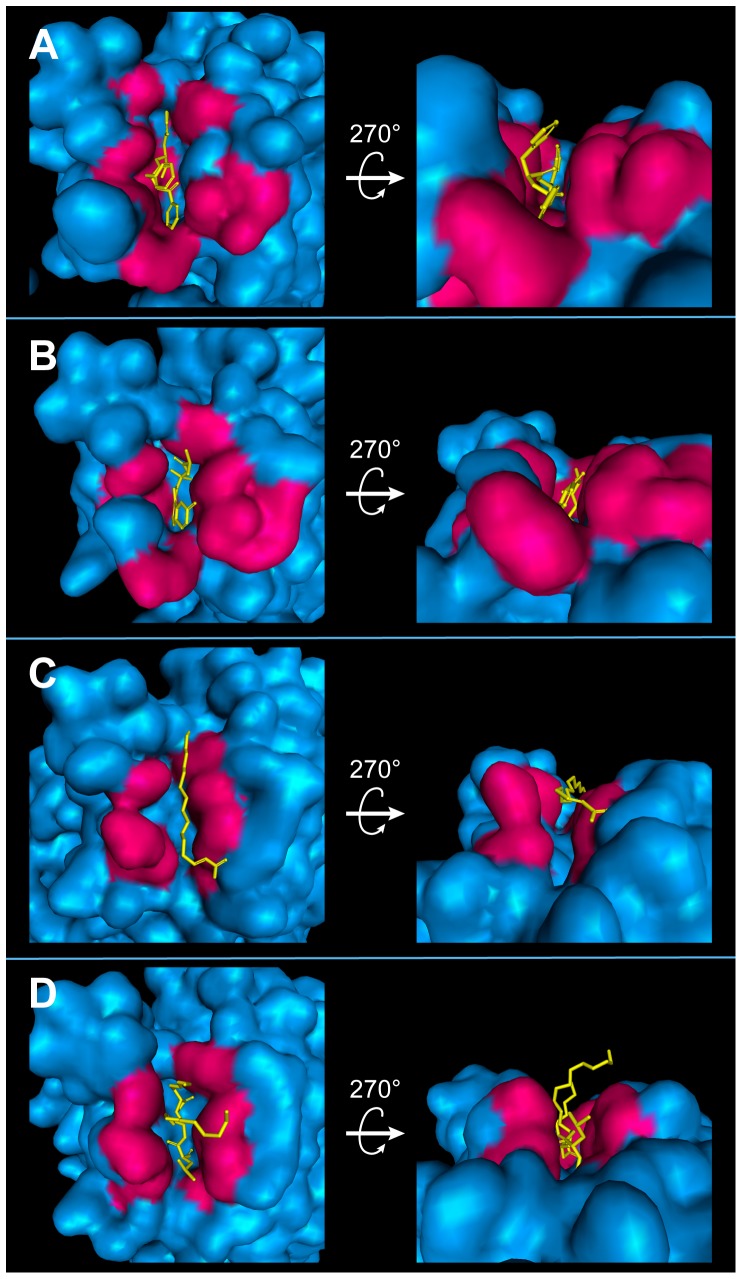
Top and side views of test compounds within the binding site following MD simulation. (A) Aurantiamide, (B) Cnidiadin, (C) 2-Hexadecenoic acid, (D) Orlistat. (D) Orlistat. Residues involved in protein-ligand interactions are shown with pink surfaces, ligands are shown in yellow.

**Figure 12:**
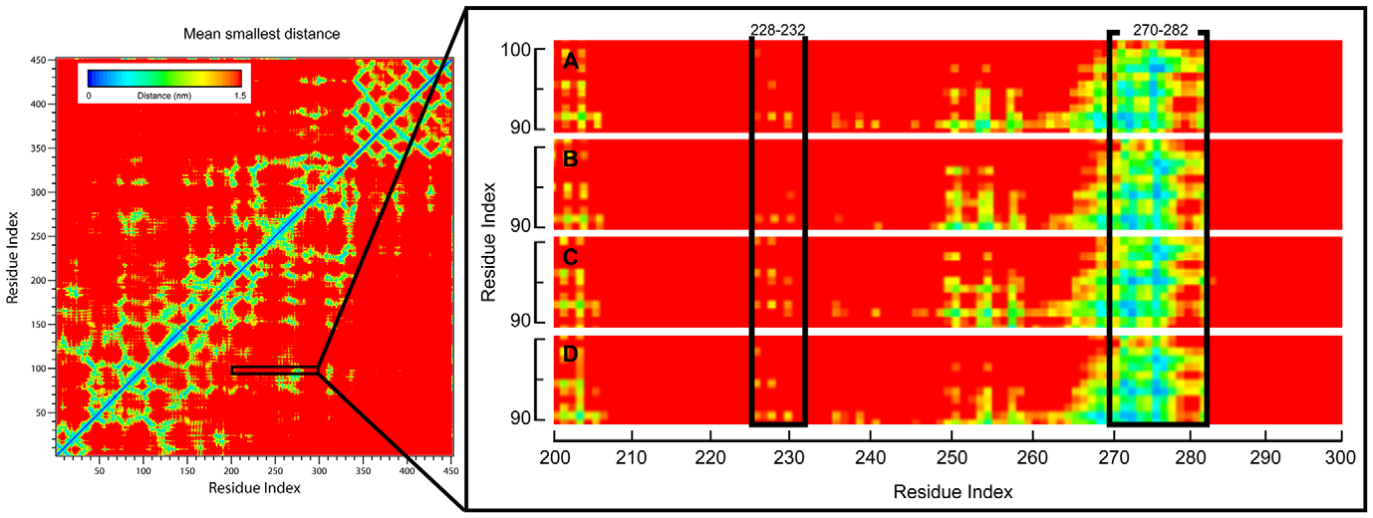
Mean smallest residue distances for individual residues using 40 ns MD conformations. Residues located in the active site cleft are shown in brackets within the enlarged illustration. (A) Aurantiamide, (B) Cnidiadin, (C) 2-Hexadecenoic acid, (D) Orlistat.

As a final validation, the positions of our test compounds in relation to the binding site after MD were evaluated for insights into potential mechanisms of inhibition (Figure 11). Interaction sites (as depicted in pink) indicate that Aurantiamide (Figure 11A), Cnidiadin (Figure 11B), and Orlistat (Figure 11D) interacted with residues on the inner regions of the cleft, and were buried deeper within the cleft. These points of interaction are neighboring to the key residue Ser169, implying possible competitive binding mechanisms. In contrast, 2-hexadecenoic acid was stabilized on the surface opening of the cleft region (Figure 11C) by the hydrophobic contacts shown in Figure 8C. This suggests that 2-hexadecanoic acid may function through blocking accessibility of the binding site. Mean smallest residue distances (Figure 12) support these speculations. 2-Hexadecenoic acid showed smaller distances between residues on opposing sides of the binding cleft. Alternatively, the larger distances measured for Aurantiamide, Cnidiadin, and Orlistat suggest that the ligands were inserted into the cleft.

## Conclusions

**Figure 13:**
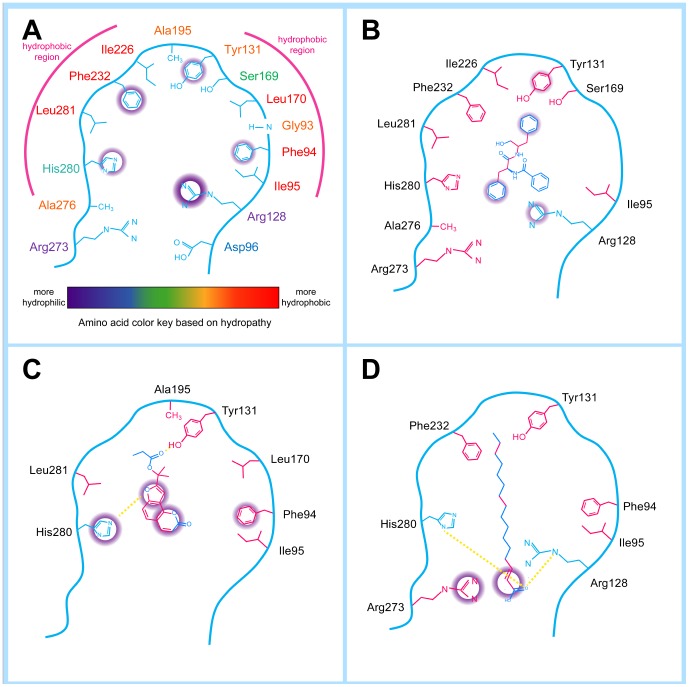
Feature summary of the PNLIP binding site and TCM residues. (A) PNLIP binding site consists of two parallel hydrophobic regions and several residues for pi-interactions as depicted by the violet rings. Amino acid legends are colored according to hydropathy. (B–D) Ligand features of (B) Aurantiamide, (C) Cnidiadin, and (D) 2-Hexadecenoic acid in coordination to binding site features are shown. Amino acids residues and ligand moieties forming hydrophobic contacts are shown in pink. Residues are moieties involved in pi-interaction are shown in violet. H-bonds are illustrated by yellow dashed lines.

Key features of the PNLIP are summarized in Figure 13A. The binding site consists of two parallel hydrophobic “walls” and five residues for potential pi-interaction formation. The hydrophobic corridor is of significance in forming a stable complex. A suitable PNLIP candidate should have structural features that correspond to those for the binding site. The cartoon diagrams of the TCM candidates (Figure 13B–13D) in PNLIP show common features of aliphatic regions for stable interactions with the hydrophobic corridor and anchoring through pi-interactions or H-bonds. Tyr131 and His280 are critical residues along with amino acids comprising the hydrophobic corridor. Integrating the results from SBDD and LBDD, we propose that the TCM candidates Aurantiamide, Cnidiadin, and 2-hexadecanoid acid have comparable drug-like characteristics to Orlistat, and may be starting points for designing novel PNLIP inhibitors for weight-control. Further investigation of these novel TCM candidates should be conducted to elucidate their possible side effects.

## Materials and Methods

### Software

Screening and molecular dynamics (MD) simulation were conducted using Discovery Studio Client v2.5.0.9164 (DS2.5; Accelrys Inc., San Diego, CA). Ligands used for docking and screening were downloaded from TCM Database@Taiwan. MATLAB (The Mathworks Inc., Natick, MA) and LibSVM [24] were used to construct MLR and SVM bioactivity prediction models. Protein-ligand interactions were analyzed with Ligplot [25].

### Docking and screening

The 3D-structure of PNLIP was downloaded from Protein Data Bank (PDB ID: 1LPB). Based on the crystal structure, the binding site of PNLIP was located near Ser169 [26], and this binding site was defined as the binding site used in this study. TCM ligands downloaded from TCM Database@Taiwan were docked and screened against the PNLIP binding site. Monte Carlo algorithm was adopted in the LigandFit module of DS2.5 for screening based on structural compatability of the ligands with the binding site. Chemistry at HARvard Molecular Mechanics (CHARMm) [27] was applied as the forcefield throughout the entire process. Docking results were ranked according to Dock Score. The commercial weight-loss drug Orlistat was selected as the control in this study. All compounds were subjected to Lipinski's Rule of Five [28]and ADMET [29]analysis for their pharmacokinetic characteristics.

### Bioactivity prediction using multiple linear regression (MLR) and support vector machine (SVM)

The 53 PNLIP inhibitors used to build bioactivity prediction models were adapted from [30] and randomly assigned to the training and test groups. All compounds were drawn with ChemBioOffice 2008 (PerkinElmer Inc., Cambridge, MA) and then ionized to physiological ionization states using the Prepare Ligand module. In addition, all experimental bioactivity values (IC_50_) were converted to logarithm values (pIC_50_). Molecular descriptors for each individual compound was calculated using Calculate Molecular Properties module, and the overall representative genetic descriptors from the pool of molecular descriptors were determined by GFA [31]. The representative genetic descriptors were applied to construct linear MLR [32] and nonlinear SVM [33] quantitative structure-activity relationship (QSAR) models using MATLAB and LibSVM [24], respectively.

The MLR model was built by MATLAB using the representative genetic descriptors is expressed as **[1]**:
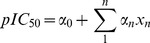
(1)α_0_ is a constant value and α_n_ is the coefficient value of descriptor *X*_n_.

Validation of the generated MLR model was conducted through cross-validation and independent tests. Robustness of the model was verified by the square correlation coefficient (R^2^) calculated between observed (experimental) pIC_50_s recorded in [30] and predicted pIC_50_ values of the training set.

SVM are supervised methods that utilize nonlinear algorithms to categorize hard-to separate patterns [34]. Utilizing an ε-insensitive loss function [35], SVM was adopted for regression (SVMR) where a function *f(x)* is identified where all training points deviate a maximum of ε from experimental values [36]. Lagrange multipliers and kernels are introduced to map input patterns into a higher dimension space **[2]**:
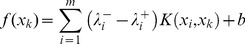
(2)where
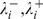
are Lagrange multipliers and *K*(*x_i_*, *x_k_*) is the kernel function.

In the LibSVM program used to construct SVM models, C cost, ε, γ, kernel type, and corresponding kernel parameters are the key parameters in determining model fit. The Gaussian radial basis function kernel **[3]** was selected as the kernel for our model.
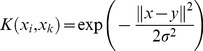
(3)Optimum C, ε, and γ values were generated by the gridregression.py command. As with MLR, cross-validation was conducted for the SVM models using default LibSVM settings. The pIC_50_ values of TCM candidates were predicted using the validated MLR and SVM models.

### Molecular dynamics (MD) simulation

Interactions between the candidate compounds and PNLIP within a dynamic system were simulated using the Molecular Dynamics module under the forcefield of CHARMm. Ligands were also prepared under default CHARMm settings. Docking poses of the TCM candidates were used as the starting structures for pre-minimization. MD simulation was conducted under vacuum conditions due to computational resource limitations and the default parallel processing restrictions in DS 2.5. Validity of the results obtained were verified by an independent MD simulation with explicit water using GROMACS (Figure S1). Energy of all complexes were minimized using 500 steps each of Steepest Descent and Conjugate Gradient. The system was heated to 310 K within 50 ps, equilibrated for 200 ps, and produced using the canonical ensemble NVT for 40 ns. SHAKE algorithm was applied to restrain bonds attached to H-atoms. During this production phase, the time steps were set at 2 fs with a temperature coupling decay time of 0.4 ps. Snapshots of the MD were taken at 20 ps intervals to analyze stability of the protein-ligand complexes.

## Supporting Information

Figure S1 **Snapshots of MD simulation by Discovery Studio (in vacuum) compared with GROMACS (with explicit water) software.** (A, C, E, G and I) were simulated by Discovery Studio, (B, D, F, H and J) were simulated by GROMACS. Following equilibrium, no difference in H-bond formation and key residues were observed between the two methods.
(TIF)
