# Supplementary figures and images for: In Silico Identification of Potent Pancreatic Triacylglycerol Lipase Inhibitors from Traditional Chinese Medicine
Source: PLoS One. 2012 Sep 6;7(9):e43932. doi: 10.1371/journal.pone.0043932 (PMC3435334; doi:10.1371/journal.pone.0043932)

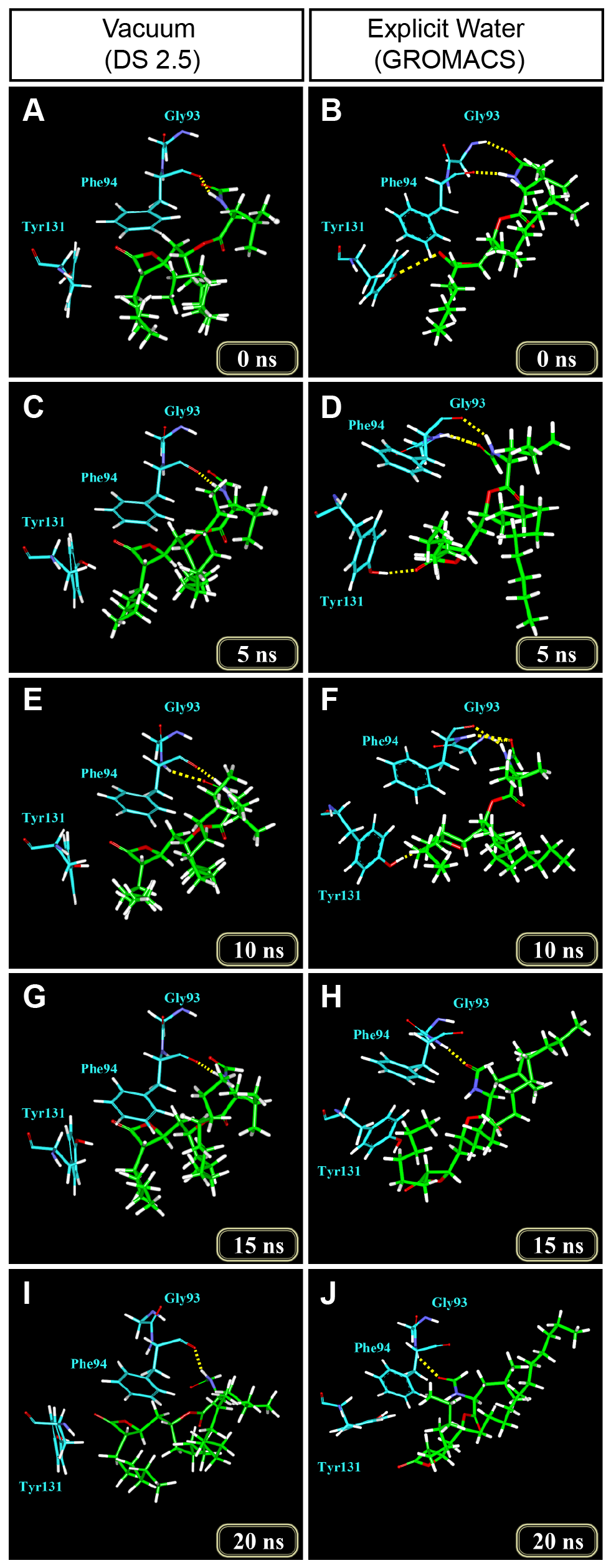

Supplement: Figure S1 — Snapshots of MD simulation by Discovery Studio (in vacuum) compared with GROMACS (with explicit water) software. (A, C, E, G and I) were simulated by Discovery Studio, (B, D, F, H and J) were simulated by GROMACS. Following equilibrium, no difference in H-bond formation and key residues were observed between the two methods. (TIF) [file pone.0043932.s001.tif]
